# A Toolkit to Facilitate the Selection and Measurement of Health Equity Indicators for Cardiovascular Disease

**DOI:** 10.5888/pcd21.240077

**Published:** 2024-10-10

**Authors:** Dorothy Wei, Simone McPherson, Refilwe Moeti, Amma Boakye, Lillian Whiting-Collins, Amena Abbas, Ebony Montgomery, Lauren Toledo, Marla Vaughan

**Affiliations:** 1 Deloitte Consulting, LLC, Atlanta, Georgia; 2 Cherokee Nation Operational Solutions, LLC, Atlanta, Georgia; 3 Centers for Disease Control and Prevention, Atlanta, Georgia; 4 ASRT, Inc, Smyrna, Georgia; 5 Veritas Management Group, Inc, Atlanta, Georgia

## Abstract

Cardiovascular disease (CVD) is the leading cause of illness and death in the US and is substantially affected by social determinants of health, such as social, economic, and environmental factors. CVD disproportionately affects groups that have been economically and socially marginalized, yet health care and public health professionals often lack tools for collecting and using data to understand and address CVD inequities among their populations of focus. The Health Equity Indicators for Cardiovascular Disease Toolkit (HEI for CVD Toolkit) seeks to address this gap by providing metrics, measurement guidance, and resources to support users collecting, measuring, and analyzing data relevant to their CVD work. The toolkit includes a conceptual framework (a visual model for understanding health inequities in CVD); a comprehensive list of health equity indicators (metrics of inequities that influence CVD prevention, care, and management); guidance in definitions, measures, and data sources; lessons learned and examples of HEI implementation; and other resources to support health equity measurement. To develop this toolkit, we performed literature scans to identify primary topics and themes relevant to addressing inequities in CVD, engaged with subject matter experts in health equity and CVD, and conducted pilot studies to understand the feasibility of gathering and analyzing data on the social determinants of health in various settings. This comprehensive development process resulted in a toolkit that can help users understand the drivers of inequities in their communities or patient populations, assess progress, evaluate intervention outcomes, and guide actions to address CVD disparities.

**Summary**

**What is already known on this topic?**

Inequities in cardiovascular disease (CVD) have resulted from historic and current systemic and structural racism and discrimination. Addressing CVD inequities requires rigorous tools to collect, measure, and interpret data.

**What is added by this report?**

We describe a step-by-step process for developing a toolkit with a suite of CVD-related health equity indicators and ways in which this measurement toolkit can advance health equity.

**What are the implications for public health practice?**

Strengthening the capacity to measure health inequities can help quantify gaps in care and develop solutions to eliminate disparities. Although our toolkit focuses on CVD, our methodology can be adapted to create measurement tools in other fields.

## Background

Cardiovascular disease (CVD), such as heart disease and stroke, is a substantial source of disability and the leading cause of death in the US, affecting nearly half of the US adult population (127.9 million people) (1). CVD death rates have increased since 2010, likely because of a rise in the prevalence of risk factors such as hypertension, diabetes, and obesity (1,2). This increase in CVD risk factors stems from worsening social, economic, and environmental conditions, which disproportionately affect some population groups (3). Disparities in CVD risk factors and outcomes across racial, ethnic, socioeconomic, and geographic groups have long been observed in the US. People living in rural areas have a higher prevalence of CVD and higher CVD death rates than urban residents (4). Additionally, Black or African American adults have the highest rate of CVD deaths and a higher prevalence of CVD risk factors, including obesity and hypertension, than other racial and ethnic groups (5). This gap in CVD outcomes is most prominent among Black and African American adults living in rural areas or areas with high levels of residential racial segregation (6).

CVD inequities are shaped by the consequences of historical and contemporary societal norms, policies, and practices that systematically reduce access to health-promoting resources (7). Structural racism, classism, sexism, and heterosexism have produced policies that created or worsened segregation of neighborhoods, schools, and work environments and fostered disinvestment in communities that have been economically and socially marginalized, leading to disparities in access to quality health care, housing, education, healthy food, employment, and opportunities for physical activity (8–11). Discriminatory practices have and continue to affect communities differentially based on race, class, or other factors, resulting in increased food insecurity, housing instability, income inequality, and unsafe and underresourced neighborhoods. Such inequitable policies and practices can create toxic stressors, which contribute to physiologic, metabolic, and psychological dysfunction that adversely affects cardiovascular health (12–16). For example, the experience of discrimination may increase the body’s acute and chronic stress response over time, leading to elevated cortisol levels, allostatic load, blood pressure, and heart rate (17–21). These increases may, in turn, lead to poor health behaviors and coping mechanisms (eg, excessive drinking, smoking, drug use) and poor mental health outcomes, all of which increase the risk for CVD (17–21).

The stark disparity in CVD mortality rates across the aforementioned factors suggests there may be benefits from focusing on health inequities in CVD prevention and management initiatives. However, health care and public health professionals often lack the tools and resources for collecting and using data to understand and address the root causes of CVD inequities. In response, the Centers for Disease Control and Prevention’s (CDC’s) Division for Heart Disease and Stroke Prevention developed a set of health equity indicators (HEIs) that measure inequities in CVD prevention, care, and management. The Health Equity Indicators for Cardiovascular Disease Toolkit (www.cdc.gov/dhdsp/health_equity/index.htm) provides measurement guidance and resources for health care and public health professionals to support the collection, measurement, and analysis of HEIs relevant to their CVD work. Toolkit users can apply HEIs to identify populations and areas of greatest need and use findings to guide policies and programs to address inequities.

The objectives of this article are to describe the methodology for selecting indicators and developing the HEI for CVD Toolkit and present its features and applications.

## Development Process

We used a multistage approach to develop the HEI for CVD Toolkit that consisted of 1) literature scans, 2) consultations with subject matter experts (SMEs), and 3) pilot studies. 

### Literature scan

**Figure 1:**
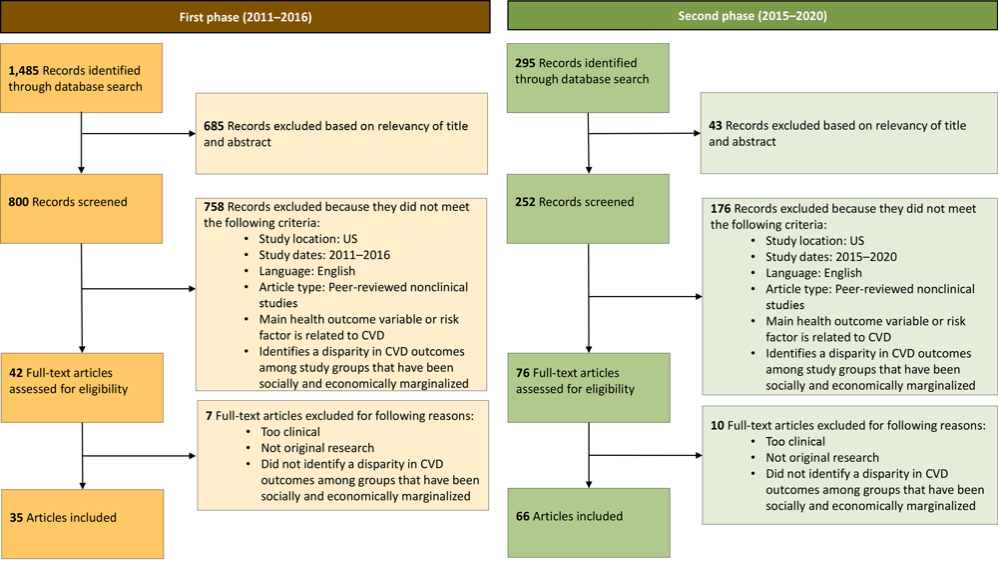
Process for conducting the literature scan for development of the Health Equity Indicators for Cardiovascular Disease Toolkit. Abbreviation: CVD, cardiovascular disease.

The literature scan aimed to identify the primary topics and themes most relevant for addressing health inequities in preventing and managing CVD. The scope of the scan focused on preventing and treating CVD and managing its risk factors. We conducted the literature scan in 2 phases: the first in 2017 and the second in 2021 to ensure inclusion of recent research and emerging trends (Figure 1).

For the first phase in 2017, we conducted a literature scan of peer-reviewed and gray literature from 2011 through 2016. We used the Ovid Medline database to search for nonclinical studies conducted in the US that identified a disparity in CVD outcomes among groups that have been economically and socially marginalized and had a primary health outcome variable that addressed CVD outcomes. The scan used the search terms *health equity* or *disparity* or *social determinants of health* and *indicators* or *framework* and *health equity* or *disparity* or *social determinants of health* and *cardiovascular disease* or *stroke.* This first scan identified 6 health equity themes, or focus areas, that correlated with poor CVD health outcomes: gender discrimination, health care access, neighborhood characteristics, racism, socioeconomic status, and stress.

**Figure 2:**
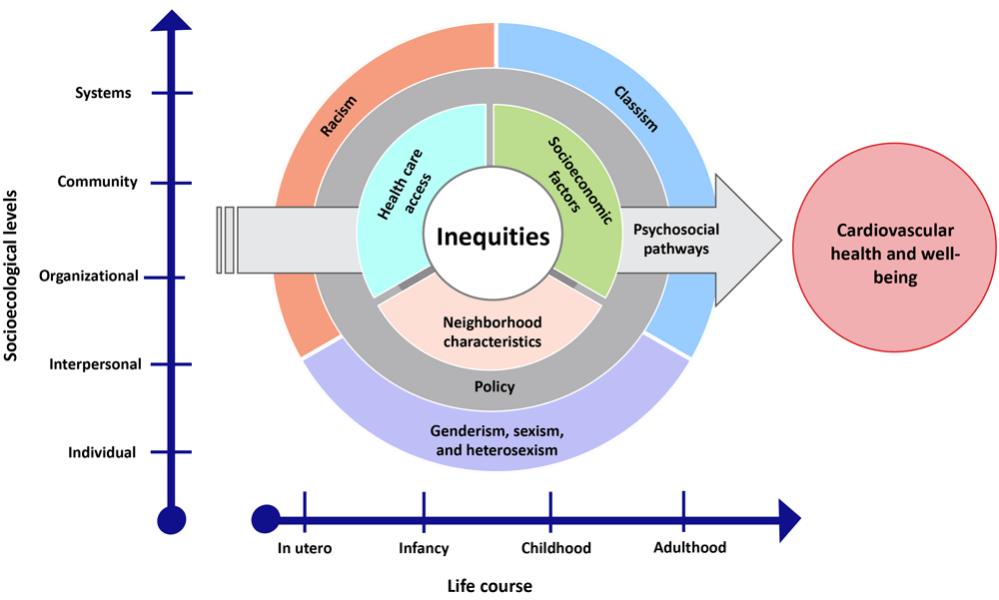
Conceptual framework for the Health Equity Indicators for Cardiovascular Disease Toolkit shows how 8 focus areas influence inequities in cardiovascular disease prevention, care, and management.

Findings from the literature scan guided the development of an initial conceptual framework, a diagram that depicts how the focus areas influence inequities pertaining to CVD outcomes and provided the structure for the development of the HEI and conceptual framework (Figure 2). For each focus area, we developed indicators to operationalize the health equity themes and measure health inequities.

For the second phase of the literature scan in 2021, we engaged 2 SMEs to verify the article review process, inclusion and exclusion criteria, and selection of final articles. We repeated the process from the first scan and updated the study window to 2015 through 2020 (Figure 1). To understand any shifts in the knowledge base on health equity since the initial scan, we compared results from both scans. This side-by-side analysis confirmed the list of 6 health equity focus areas and indicators and identified 2 additional focus areas and potential indicators. We identified and refined 8 focus areas as a result of the updated scan: genderism, sexism, and heterosexism; health care access; neighborhood characteristics; policy; psychosocial pathways; racism; socioeconomic factors; and classism. We used results from the updated literature scan to refine the conceptual framework (Figure 2) and HEIs, provide evidence on the relevance of the HEIs for addressing inequities for CVD prevention and management, and create indicator profiles that provide guidance for measuring HEIs. A strength of the literature scan approach was our 2-phase process for updating the literature to ensure inclusion of recent research and emerging trends. Additionally, expert consultations ensured the quality and relevance of selected articles. However, our method did not appraise the quality of included studies or assess the effect size as rigorously as other types of research reviews, such as systematic reviews or meta-analyses, which could have potentially introduced an element of bias into our findings.

### Engagement with SMEs

We collaborated with SMEs in health equity and CVD at multiple points in the development process to inform the toolkit’s content. First, we engaged with a panel of SMEs through a series of workshops conducted from March through July 2021. We used a participatory approach to identify SMEs and ensure that the SME panel represented diverse backgrounds. The SME panel included 13 researchers and 8 practitioners from universities, research institutions, health care organizations, and state, local, and tribal health departments. As academic experts in CVD and health equity, researcher SMEs provided technical and methodological support for HEI development. Researcher SMEs identified measures and data sources for operationalizing the HEIs, outlined measurement considerations, and validated the technical integrity and robustness of measures. Practitioner SMEs assessed the feasibility and applicability of the HEIs by using their expertise in translating research to public health practice. Practitioner SMEs also used case examples and field notes to provide practical considerations for measurement guidance in the indicator profiles and feedback on how to share experiences with health equity measurement.

After the initial SME panels, we re-engaged 2 researcher SMEs in 2022. Both SMEs verified the second literature scan, identified gaps and areas of improvement for the conceptual framework, refined the definitions and measurement guidance provided in the indicator profiles, and finalized the selection of HEIs, measures, and data sources. All final materials in the indicator profiles and conceptual framework were reviewed and approved by SMEs. The 2 SMEs also actively guided the design, implementation, and analysis of the HEI Pilot Study, which tested a subset of HEIs at various health care organizations. Specifically, they provided advice on the criteria for site selection, data collection methods, and interpretation of the HEI Pilot Study findings.

### Pilot studies

We conducted 2 pilot studies in health care and public health settings to assess the feasibility of gathering and analyzing data on a subset of HEIs. One pilot study assessed the feasibility of HEI measurement and use in health care and public health organizations (the HEI Pilot Study) and the second pilot study focused on lived experiences with CVD and the patient perspective of providing information related to social determinants of health (the Patient-Informed HEI Pilot Study).

The HEI Pilot Study, conducted from January through April 2022, was designed to understand the factors that support or hinder an organization’s data collection, measurement, and analysis of HEIs. To solicit nominations of pilot sites, we disseminated recruitment materials through the networks of our SMEs and our partnerships with health departments, health care, health equity, and cardiovascular health–focused organizations and associations. Health departments, health systems, and health care providers were eligible to participate in the pilot if they demonstrated 1) a commitment to reducing health inequities in their communities, and 2) the capacity to collect and report data. Eligible sites were selected for the pilot through a prioritization process involving a collaborative review and ranking of the information provided in the recruitment screening form. Twenty-six organizations completed the online screening form designed to assess the existing capacity of each site, including access to data, engagement in health equity interventions, populations served, settings, and geographic area. The site-selection process prioritized organizations that work in different health care settings, serve diverse populations and geographic areas, have experience collecting data for similar equity-related indicators, demonstrate capacity to collect and use data for HEIs, and are committed to or interested in health equity programming. We used a systematic indicator ranking process to select a subset of HEIs and ranked HEIs from each focus area based on feasibility and relevance, including the degree to which data for the HEI can be captured by using multiple measures and interest in the indicator among participating sites based on relevance to their organizations and access to the required data sources.

We recruited 7 organizations to pilot-test 20 HEIs and provided pilot sites with site-specific data collection guides, which specified definitions, data sources, and instructions on collecting and measuring HEIs. The HEI Pilot Study used an exploratory convergent mixed-methods design in which our team collected and analyzed quantitative and qualitative data concurrently and used one to inform the other. We used semistructured interviews and reporting forms to collect information about the data collection processes at pilot sites. We used thematic analysis, content analysis, and descriptive statistics to analyze results. Findings from this pilot study documented challenges with data collection, such as limited access to census tract data, and lack of trainings or standardized protocols to support HEI data collection. Findings also highlighted the value of organizational commitment to health equity, leadership buy-in, staff capacity, and partnerships to support health equity measurement efforts. CDC designated this work as nonresearch and exempt from institutional review board review.

The second pilot was a yearlong study conducted from August 2021 to August 2022 at an outpatient center for a public safety-net hospital in downtown Atlanta, Georgia. The purpose of the Patient-Informed HEI Pilot Study was to further assess facilitators and barriers to collecting HEIs and understand patients’ lived experiences with CVD through HEIs. The Patient-Informed HEI Pilot Study used a mixed-methods approach to collect patient data about the following focus areas: psychosocial pathways, racism, neighborhood characteristics, socioeconomic factors, and health care access. We conducted semistructured interviews via Zoom with 10 patients and collected data on patient demographic characteristics and CVD risk (ie, diabetes, hypertension, drinking and smoking behavior) from 60 patients via Healthy Planet, a population health management software within the EPIC electronic medical record system. We then used thematic analysis to analyze qualitative data and identify key drivers of inequities. We also generated descriptive statistics for patients, including the frequency of health outcomes (eg, diabetes, hypertension) and demographic characteristics (eg, age, race and ethnicity, sex and gender). Chi-square tests found a significant correlation between increased CVD risk and increased neighborhood-level social vulnerability (ie, the potential adverse effects on communities that result from external stresses on human health) (22). Clinician interviews highlighted barriers and facilitators to data collection. Barriers included incomplete data on the social determinants of health, and facilitators included leadership support and trusting relationships between staff and patients. Clinician interviewees suggested that to address the barrier of incomplete data, a standardized intake procedure could be implemented to routinely collect data on the social determinants of health when new patients visit the outpatient center. This study helped to establish a deep understanding of patients living with CVD in an outpatient setting, and findings were used to inform and strengthen ongoing quality improvement for health equity.

Findings from the pilot studies were used to update and clarify the guidance provided in the indicator profiles (eg, additional instructions on data availability, accessing data sources, and calculating measures, when applicable) and develop case examples that illustrate the real-world application of HEIs to inform health equity efforts. This pilot study was approved by the institutional review board of the Grady Research Oversight Committee.

## Application

### Key features and content

**Figure 3:**
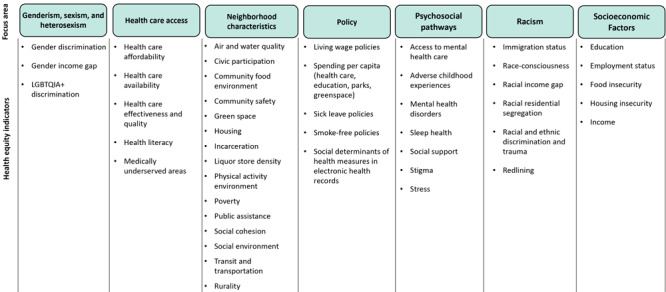
List of health equities indicators (HEIs), by focus area, in the Health Equity Indicators for Cardiovascular Disease Toolkit. The list of HEIs was confirmed by a literature review and consultation with subject matter experts. Abbreviation: LGBTQIA+, lesbian, gay, bisexual, transgender, queer, intersex, asexual, and other sexual orientation and gender identity populations.

The toolkit is an easy-to-navigate website that features a conceptual framework, a set of HEIs, profiles with measurement details for each indicator, case examples, field notes, resources, and a glossary of terms. The HEIs are measurable constructs that represent multiple health equity focus areas influencing CVD prevention, care, and management (Figure 3). The selection of HEIs was guided by the conceptual framework (Figure 2).

The conceptual framework is based on the socioecological model (23). The framework provides a model for understanding health inequities in CVD by visually representing how the 8 focus areas span structural and socioenvironmental drivers across socioecological levels and throughout the lifespan to influence cardiovascular health (24). In the framework, the health equity focus areas overlap each other as nested circles to illustrate that these areas are interconnected and can occur across all stages of life (from in utero to adulthood) and all socioecological levels (from individual to systems level). The innermost layer of the circle is labeled “inequities” and an arrow cuts across the nested layers and points toward “Cardiovascular health and well-being” to illustrate how cardiovascular health and well-being are affected by inequities across focus areas. 

Indicator profiles are measurement guidance documents that describe the relevance of the indicators and provide the definitions, measures, and data sources for each HEI (https://www.cdc.gov/dhdsp/health_equity/profiles.htm). Some indicators may have more than 1 measure to assess various attributes of the indicator. The indicator profiles provide guidance on accessing existing data sources for secondary data collection and survey instruments for primary data collection for each measure. Most data sources are publicly available, easy to access, and free to users. In total, the HEI for CVD Toolkit presents 46 HEIs, 112 measures, and 245 data sources. The list of indicators does not cover all constructs for CVD-related health equity and will surely evolve as information on health equity measurement advances. Therefore, this toolkit is a living document and will be updated with new information as the literature progresses. Still, users seeking to incorporate health equity measurement in their work can consider this toolkit as a starting point.

The pilot studies resulted in examples of how HEIs were used by pilot sites. These case examples document the experiences of health care and public health organizations in collecting and analyzing data, and describe barriers, facilitators, and lessons learned. We also gathered additional examples of health equity measurement and describe how health departments use HEIs to develop programs and activities. We gathered these examples from practitioner SMEs, not from the pilot studies, and compiled them into field notes.

The toolkit’s resource page includes various resources available from CDC and other organizations, including toolkits, guides, research articles, infographics, and screening tools. These resources can help to support health equity measurement and evaluation and guide health care organizations to advance their work in addressing health disparities. Lastly, the HEI for CVD Toolkit includes a glossary of terms to define frequently used terms and concepts.

### Using the toolkit to select and measure health inequities

The toolkit is intended to inform the work of health care and public health professionals and organizations in reducing disparities in CVD outcomes, including state and local health departments, health care professionals and organizations, nonprofit organizations, clinicians, researchers, and policymakers. It can support users across various functions, including research, program evaluation, program planning, and policy development. For example, HEIs can be used to learn about the socioeconomic conditions of a population of interest and monitor changes over time, assess progress in efforts to reduce health inequities, and evaluate program outcomes. These findings can then help to identify populations and areas of greatest need and determine program successes and gaps, which can inform future policies and programs. We describe a hypothetical scenario of how a toolkit user from a nonprofit organization might select, measure, and use an HEI to inform their work and advance health equity (Table).

**Table:** Sample Scenario for Application of the Health Equity Indicators for Cardiovascular Disease Toolkit by a Nonprofit Organization

Question	Toolkit application
What problem can I solve by using the HEI Toolkit for CVD?	How to expand a housing assistance program and understand its impact on CVD in a community
Which HEI should I use?	Use the housing insecurity indicator in the socioeconomic factors focus area and select the housing instability measure.
What can I learn from the indicator profile?	People experiencing unstable housing or housing insecurity and people who are not securely housed are more likely to delay medical care and use emergency care, have poorer health care access, experience adverse mental health outcomes, and have a higher prevalence of substance use compared with people who have stable housing (14,25,26). Housing insecurity can be linked to CVD risk and related mortality due to downstream consequences of psychological distress and competing stressors (ie, spending on housing rather than medical care).
How can I apply this HEI to my work?	· Use the US Department of Housing and Urban Development Comprehensive Housing Affordability Strategy query tool to extract data on the number of households experiencing 1 or more housing issues (eg, overcrowding, paying more than 50% of income on housing costs, inadequate facilities) in their city. · Assess the relationship between housing insecurity and CVD outcomes using correlation, geospatial, or regression analysis.
How can this analysis advance health equity?	Apply findings in a grant application to expand program operations and/or use findings to guide efforts to increase affordable housing development.
What other information can I find in the HEI Toolkit for CVD?	· *Case Examples and Field Notes* are included throughout the Health Equity Indicator Profiles. The Case Examples and Field Notes include short summaries that describe an organization’s experience with gathering data for specific indicators and lessons learned. · *Related Resources Page* **: ** www.cdc.gov/dhdsp/health_equity/resources.htm. This web page provides additional information that supports health equity measurement and evaluation, guides health care organizations to advance their health equity work, and helps health care organizations address health disparities. For each resource, a title, URL, author, and description is given.

Abbreviations: CVD, cardiovascular disease; HEI, health equity indicator.

## Conclusion

We developed the HEI for CVD Toolkit by using a robust approach to address the gap between the immediate need for tools to measure and evaluate CVD inequities and the limited availability of a comprehensive and cohesive resource. This multistage process produced a toolkit to improve the capacity of health care and public health professionals to measure health inequities, establish goals, and track progress in achieving equity. Although the toolkit focuses on CVD, the methodology can be adapted to create measurement tools in other public health programs. The toolkit includes various measures to evaluate diverse interventions that address the root causes of health inequities and assess progress in reducing health disparities among groups that have been socially or economically marginalized.

## References

[1] Tsao CW , Aday AW , Almarzooq ZI , Anderson CAM , Arora P , Avery CL , ; American Heart Association Council on Epidemiology and Prevention Statistics Committee and Stroke Statistics Subcommittee. Heart disease and stroke statistics — 2023 update: a report from the American Heart Association. *Circulation.* 2023;147(8):e93–e621. 10.1161/CIR.0000000000001123 36695182 PMC12135016

[2] Sidney S , Go AS , Jaffe MG , Solomon MD , Ambrosy AP , Rana JS . Association between aging of the US population and heart disease mortality from 2011 to 2017. *JAMA Cardiol.* 2019;4(12):1280–1286. 10.1001/jamacardio.2019.4187 31663094 PMC6822092

[3] Harris KM , Woolf SH , Gaskin DJ . High and rising working-age mortality in the US: a report from the National Academies of Sciences, Engineering, and Medicine. *JAMA.* 2021;325(20):2045–2046. 10.1001/jama.2021.4073 33970196

[4] Harrington RA , Califf RM , Balamurugan A , Brown N , Benjamin RM , Braund WE , . Call to action: rural health: a presidential advisory from the American Heart Association and American Stroke Association. *Circulation.* 2020;141(10):e615–e644. 10.1161/CIR.0000000000000753 32078375

[5] National Center for Health Statistics. *Racial and Ethnic Disparities in Heart Disease.* April 2019. Accessed May 30, 2023. https://www.cdc.gov/nchs/hus/spotlight/HeartDiseaseSpotlight_2019_0404.pdf

[6] Kyalwazi AN , Loccoh EC , Brewer LC , Ofili EO , Xu J , Song Y , . Disparities in cardiovascular mortality between Black and White adults in the United States, 1999 to 2019. *Circulation.* 2022;146(3):211–228. 10.1161/CIRCULATIONAHA.122.060199 35861764 PMC9310198

[7] Weinstein JN , Geller A , Negussie Y , Baciu A , eds. The root causes of health inequity. In: *Communities in Action: Pathways to Health Equity.* National Academies of Sciences, Engineering, and Medicine; Health and Medicine Division; Board on Population Health and Public Health Practice; Committee on Community-Based Solutions to Promote Health Equity in the United States; 2017. Accessed May 30, 2023. https://www.ncbi.nlm.nih.gov/books/NBK425845 28418632

[8] Powell-Wiley TM , Baumer Y , Baah FO , Baez AS , Farmer N , Mahlobo CT , . Social determinants of cardiovascular disease. *Circ Res.* 2022;130(5):782–799. 10.1161/CIRCRESAHA.121.319811 35239404 PMC8893132

[9] Williams DR , Lawrence JA , Davis BA . Racism and health: evidence and needed research. *Annu Rev Public Health.* 2019;40(1):105–125. 10.1146/annurev-publhealth-040218-043750 30601726 PMC6532402

[10] Homan P . Structural sexism and health in the United States: a new perspective on health inequality and the gender system. *Am Sociol Rev.* 2019;84(3):486–516. 10.1177/0003122419848723

[11] Institute of Medicine (US) Committee on Lesbian, Gay, Bisexual, and Transgender Health Issues and Research Gaps and Opportunities. *The Health of Lesbian, Gay, Bisexual, and Transgender People: Building a Foundation for Better Understanding.* National Academies Press; 2011. Accessed May 30, 2023. http://www.ncbi.nlm.nih.gov/books/NBK64806/ 22013611

[12] Diez Roux AV , Mujahid MS , Hirsch JA , Moore K , Moore LV . The impact of neighborhoods on CV risk. *Glob Heart.* 2016;11(3):353–363. 10.1016/j.gheart.2016.08.002 27741982 PMC5098701

[13] Claudel SE , Adu-Brimpong J , Banks A , Ayers C , Albert MA , Das SR , . Association between neighborhood-level socioeconomic deprivation and incident hypertension: a longitudinal analysis of data from the Dallas heart study. *Am Heart J.* 2018;204:109–118. 10.1016/j.ahj.2018.07.005 30092412 PMC6217793

[14] Sims M , Kershaw KN , Breathett K , Jackson EA , Lewis LM , Mujahid MS , ; American Heart Association Council on Epidemiology and Prevention and Council on Quality of Care and Outcomes Research. Importance of housing and cardiovascular health and well-being: a scientific statement from the American Heart Association. *Circ Cardiovasc Qual Outcomes.* 2020;13(8):e000089. 10.1161/HCQ.0000000000000089 32673512 PMC7442620

[15] Krieger N . Measures of racism, sexism, heterosexism, and gender binarism for health equity research: from structural injustice to embodied harm — an ecosocial analysis. *Annu Rev Public Health.* 2020;41(1):37–62. 10.1146/annurev-publhealth-040119-094017 31765272

[16] Laraia BA . Food insecurity and chronic disease. *Adv Nutr.* 2013;4(2):203–212. 10.3945/an.112.003277 23493536 PMC3649100

[17] Caceres BA , Brody A , Luscombe RE , Primiano JE , Marusca P , Sitts EM , . A systematic review of cardiovascular disease in sexual minorities. *Am J Public Health.* 2017;107(4):e13–e21. 10.2105/AJPH.2016.303630 28207331 PMC5343694

[18] Molix L . Sex differences in cardiovascular health: does sexism influence women’s health? *Am J Med Sci.* 2014;348(2):153–155. 10.1097/MAJ.0000000000000300 25054736 PMC4111152

[19] Lewis TT , Williams DR , Tamene M , Clark CR . Self-reported experiences of discrimination and cardiovascular disease. *Curr Cardiovasc Risk Rep.* 2014;8(1):365. 10.1007/s12170-013-0365-2 24729825 PMC3980947

[20] Williams DR , Mohammed SA . Discrimination and racial disparities in health: evidence and needed research. *J Behav Med.* 2009;32(1):20–47. 10.1007/s10865-008-9185-0 19030981 PMC2821669

[21] Geronimus AT , Hicken M , Keene D , Bound J . “Weathering” and age patterns of allostatic load scores among Blacks and Whites in the United States. *Am J Public Health.* 2006;96(5):826–833. 10.2105/AJPH.2004.060749 16380565 PMC1470581

[22] Agency for Toxic Substances and Disease Registry. CDC/ATSDR Social Vulnerability Index (SVI): overview. Published November 16, 2022. Last reviewed June 14, 2024. Accessed June 16, 2023. https://www.atsdr.cdc.gov/placeandhealth/svi/index.html

[23] National Center for Injury Prevention and Control, Division of Violence Prevention. About violence prevention: a framework for prevention. Published January 18, 2022. Accessed June 16, 2023. https://www.cdc.gov/violence-prevention/about/index.html

[24] Bronfenbrenner U . Toward an experimental ecology of human development. *Am Psychol.* 1977;32(7):513–531. 10.1037/0003-066X.32.7.513

[25] Taylor LA . Housing and health: an overview of the literature. *Health Affairs Health Policy Brief.* Published online June 7, 2018. Accessed May 30, 2023. https://www.healthaffairs.org/content/briefs/housing-and-health-overview-literature

[26] D’Alessandro D , Appolloni L . Housing and health: an overview. *Ann Ig.* 2020;32(5 Suppl 1):17–26. 33146364 10.7416/ai.2020.3391

